# Statistical measures for defining an individual's degree of independence within state-dependent dynamic games

**DOI:** 10.1186/1471-2148-6-81

**Published:** 2006-10-12

**Authors:** Sean A Rands, Rufus A Johnstone

**Affiliations:** 1 Department of Zoology, University of Cambridge, Downing Street, Cambridge CB2 3EJ, UK; 2 Centre for Ecology and Conservation, University of Exeter in Cornwall, Tremough Campus, Penryn, Cornwall TR10 9EZ, UK; 3 Centre for Behavioural Biology, Department of Clinical Veterinary Science, University of Bristol, Langford House, Langford, North Somerset BS40 5DU, UK

## Abstract

**Background:**

For organisms living or interacting in groups, the decision-making processes of an individual may be based upon aspects of both its own state and the states of other organisms around it. Much research has sought to determine how group decisions are made, and whether some individuals are more likely to influence these decisions than others. State-dependent modelling techniques are a powerful tool for exploring group decision-making processes, but analyses conducted so far have lacked methods for identifying how dependent an individual's actions are on the rest of the group.

**Results:**

Here, we introduce and evaluate two easy-to-calculate statistics that quantify how dependent an individual's actions are upon the state of a co-player in a two-player state-dependent dynamic game. We discuss the merits of these statistics, and situations in which they would be useful.

**Conclusion:**

Our statistical measures provide a means of quantifying how independent an individual's actions are. They also allow researchers to quantify the output of state-dependent dynamic games, and quantitatively assess the predictions of these models.

## Background

The development of state-dependent modelling techniques applicable to evolutionary biology has allowed us to address many questions about the behaviour and life history strategies of organisms. Behavioural or life history decisions induce changes in the state of the organism (where state can represent physical aspects of the organism, such as its energy reserves, or other changeable properties of the individual, such as its number of successful matings) [1,2]. These changes in the organism's state are then linked to changes in some measure of its fitness (such as lifetime reproductive success). This means that state-dependent models use an evolutionarily meaningful currency [3]. As well as being used to generate testable predictions about the behaviour of individuals (see [4-8] for some examples of successful tests), these state-dependent techniques have also been extended to consider interactions between individuals, using game-theoretic techniques. For example, state-dependent dynamic games have examined competition between animals over resources [9], games between predators and prey [10], signalling [11-14], mate choice [15], social foraging [16-19], aggregation behaviour [20], and parental care [21-23].

In evolutionary and behavioural ecology, one area that has been the focus of a great deal of attention recently is how groups of animals come to make combined decisions (behaviours by individuals within a group that are dependent upon belonging to the group, and which may have effects upon both themselves and other individuals within the group) [17,24]. State-dependent dynamic games are an important theoretical tool for examining decision-making processes by the individuals within a group, where the inclusion of state components allows us to add a degree of complexity and realism that is lacking from state-free game theory (e.g. [25,26]). However, although state-dependent dynamic games are extremely powerful in the range of predictions that they are able to make, they can be difficult to analyse. In most of the cases listed above, the models are solved numerically rather than analytically. This usually means that sensitivity analysis then has to be conducted using a wide range of parameter values, and the results of these analyses have to be summarised in some manner that gives insight into the behaviour of the model. This can be done by visually comparing the policies generated for meaningful trends (see, for example, [21,22]), or by using the policies generated to simulate the behaviour of individuals within a population, and then comparing these predicted behaviours in response to different parameter values ([17] gives an example where both policies and population behaviours are examined).

When we look at group decision-making strategies, it is important for us to know how decisions are being made, and who is making them. It is possible to explore this using dynamic game models. For example, Rands *et al*. [17] developed a game in which a pair of individuals following identical state-dependent strategies spontaneously separate into different decision-making rôles based upon random fluctuations in their state (one member of the pair becomes the 'leader', initiating all changes in behaviour, whilst the other 'follower' individual copies its actions). These rôles can be defined more rigorously by reference to the degree of independence that an individual shows in its actions: in the model described in [17], we can define the 'leader' as the 'individual whose actions are less dependent upon the actions and/or state of its co-player', and the 'follower' as the 'individual whose actions are more dependent upon the actions and/or state of its co-player' (Note that we use 'leader' here specifically to refer to the individual whose actions are more likely to determine the combined actions of the pair – by using the term, we do not imply any other special properties of the individuals such as dominance. See [27] for further discussion of leadership terminology.) In [17], it was relatively straightforward to assess which individual would become the 'leader', because the policies generated were not difficult to interpret. However, more complex models are very likely to throw up policies that are less easy to decipher.

The potential complexity of the results generated by state-dependent dynamic games needn't be a barrier to using them as tools for investigating group decision-making and social behaviour, provided we are able to develop techniques for quantifying and comparing pertinent aspects of the models, such as the degree of autonomy different individuals have in determining their behaviour. In this paper, we develop two statistics for quantifying the degree of independence that individuals show in a two-player game. Using these statistics give us a new tool for investigating decision-making processes using state-dependent dynamic games.

## Results and discussion

To generate our independence statistics, we first assume that the solution has been found to a game between two players. Within this game, each animal in the pair performs a behaviour at a given moment in time. For simplicity, we assume here that a behaviour is the probability of performing one of two possible actions, and we refer to this behaviour as the 'principal action'. As an example, the model developed in [17] examined individuals who could either forage or rest, and so if we define 'rest' as the principal behaviour, if *p*_*xy *_= 0.25 for a pair of players in states *x*, *y*, the focal player should rest 25% of the time, and forage 75% of the time (the chance of conducting the principal action is defined with a continuously-distributed percentage here, rather than being limited to 0% or 100%, as we are assuming that the behaviours defined in our policy are of the 'responses with error' form defined by McNamara *et al*. [28]). Note that although the statistics we derive are dependent upon the distributions of states and responses to those states in a two-player example, they could easily be extended to consider multi-player games.

In the game, both players possess a changeable quality that is defined by a state variable. For convenience, we define the state variable of the focal player as *x*, and that of its co-player as *y*. The solution to the game defines a behavioural response (usually taken to be the optimal response, although this is not an essential condition for the statistics derived here) for each player based on the current state value of both itself and its co-player, and so for a focal player in state *x *with co-player in state *y*, its action is *p*_*xy *_as defined by the game's solution. We refer to the full set of behavioural responses for all possible combinations of state pairs as the 'policy' of an individual. For simplicity, we assume that there is no dependence upon time in this example, meaning that the players are following the same policy at each decision period, and their actions are only dependent upon the current state of their co-player (note that the techniques described could be extended to consider time, if time is included as a state-variable). Note also that the statistic we derive is independent of the measures of fitness used to derive the policy (in order to assess the effects of decision making upon fitness, it is necessary to consider the canonical costs of making errors, as discussed in [3]).

We also assume that the expected state distribution of a stable population has been determined, for instance by forward-running a population of player pairs following the calculated policy using a Markov-chain process [2], or by calculation [1]. We therefore know that for any state pair *x*, *y*, the proportion of the current population in that state pair is *d*_*xy*_, where ∑_*x*,*y*_*d*_*xy *_= 1.

### Statistic 1: C, based upon the absolute size of a mistake made when estimating behaviour

Our first statistic considers a situation where the focal individual knows its own state *x*, but does not know the state of its co-player *y*. Although the focal knows its own state, its policy may define a large range of possible behaviours in response to the possible states of its co-player. Although exact information about the co-player's state doesn't exist, it is possible to calculate the probability that the co-player is in each state *y *because we have already calculated the expected state-distribution of the population. Therefore, we can calculate *g*_*x*_, a 'best guess' as to the behaviour to conduct based upon weighting the policy-determined behaviours for all possible co-player states by the probabilities that the co-player is in those states (note that this 'best guess' is not equivalent to the optimal behaviour in the absence of information about the co-player's state, since the costs of making errors are not taken into account). Therefore, this best guess represents the behaviour that maximises the proportion of time that the focal individual in any state *x *responds in the appropriate manner (as defined by its policy) to a co-player in state *y*, despite the focal player not being able to assess *y*.

Based on the likelihood of the pair being in states *x*, *y*, the best guess is calculated as

*g*_*x *_= ∑_*y*_(*p*_*xy*_*d*_*xy*_)/∑_*y*_*d*_*xy*_.     (1)

The statistic *C *quantifies the 'incorrectness' of this best guess. For a state pair *x*, *y*, the 'incorrectness' of a best guess is given by

|*g*_*x *_- *p*_*xy*_|.     (2)

For a focal individual in state *x*, the size of the worst mistake it can therefore make is the maximum value of *g*_*x *_and 1 - *g*_*x*_. Therefore, recalculating eqn. **2 **as

|gx−pxy|max⁡(gx,1−gx)     (3)
 MathType@MTEF@5@5@+=feaafiart1ev1aaatCvAUfKttLearuWrP9MDH5MBPbIqV92AaeXatLxBI9gBaebbnrfifHhDYfgasaacH8akY=wiFfYdH8Gipec8Eeeu0xXdbba9frFj0=OqFfea0dXdd9vqai=hGuQ8kuc9pgc9s8qqaq=dirpe0xb9q8qiLsFr0=vr0=vr0dc8meaabaqaciaacaGaaeqabaqabeGadaaakeaadaWcaaqaamaaemaabaGaem4zaC2aaSbaaSqaaiabdIha4bqabaGccqGHsislcqWGWbaCdaWgaaWcbaGaemiEaGNaemyEaKhabeaaaOGaay5bSlaawIa7aaqaaiGbc2gaTjabcggaHjabcIha4naabmaabaGaem4zaC2aaSbaaSqaaiabdIha4bqabaGccqGGSaalcqaIXaqmcqGHsislcqWGNbWzdaWgaaWcbaGaemiEaGhabeaaaOGaayjkaiaawMcaaaaacaWLjaGaaCzcamaabmaabaGaeG4mamdacaGLOaGaayzkaaaaaa@4A9D@

gives us the size of the mistake made at *x*, *y *relative to the worst mistake possible. For a given value of *x*, we can sum this over all values of *y *(adjusted accordingly for the population distribution) to calculate the severity of the mistake made when the focal individual is in state *x*. In this way, we can calculate an overall incorrectness statistic *C*, considering all possible state pairs and their distributions, as

C=∑x,y(|gx−pxy|max⁡(gx,1−gx))dxy.     (4)
 MathType@MTEF@5@5@+=feaafiart1ev1aaatCvAUfKttLearuWrP9MDH5MBPbIqV92AaeXatLxBI9gBaebbnrfifHhDYfgasaacH8akY=wiFfYdH8Gipec8Eeeu0xXdbba9frFj0=OqFfea0dXdd9vqai=hGuQ8kuc9pgc9s8qqaq=dirpe0xb9q8qiLsFr0=vr0=vr0dc8meaabaqaciaacaGaaeqabaqabeGadaaakeaacqWGdbWqcqGH9aqpdaaeqaqaamaabmaabaWaaSaaaeaadaabdaqaaiabdEgaNnaaBaaaleaacqWG4baEaeqaaOGaeyOeI0IaemiCaa3aaSbaaSqaaiabdIha4jabdMha5bqabaaakiaawEa7caGLiWoaaeaacyGGTbqBcqGGHbqycqGG4baEdaqadaqaaiabdEgaNnaaBaaaleaacqWG4baEaeqaaOGaeiilaWIaeGymaeJaeyOeI0Iaem4zaC2aaSbaaSqaaiabdIha4bqabaaakiaawIcacaGLPaaaaaaacaGLOaGaayzkaaGaemizaq2aaSbaaSqaaiabdIha4jabdMha5bqabaaabaGaemiEaGNaeiilaWIaemyEaKhabeqdcqGHris5aOGaeiOla4IaaCzcaiaaxMaadaqadaqaaiabisda0aGaayjkaiaawMcaaaaa@5948@

*C *defines the degree of independence an individual has in its actions. If *C *= 0, then even when the co-player's state is unknown, the focal player's 'best guess' as to the action specified by its policy is always correct. This implies that its actions are completely independent of the co-player's state. By contrast, if *C *= 1, the focal individual's best guess is no better than if it were to make the worst mistake possible, implying that its actions are maximally dependent upon the state of the co-player.

The necessity of using eqn. **3 **instead of eqn. **2 **becomes apparent with a few examples. Consider a state distribution in a population of pairs in which the focal players are all in the same state, whilst 50% of the co-players are in state 1, and the other 50% are in state 2. If we represent the state-dependent policy set of the focal individual as *p*_*y *_= {*b*_1_, *b*_2_} where *b*_*i *_is the likelihood of performing a set behaviour if the co-player is in state *i*, then it is straightforward to show that if *p*_*y *_= {0,0} or *p*_*y *_= {1, 1}, then *C *= 0, meaning that the actions of the focal player are not dependent upon the state of its co-player. If we consider an adjusted version of the incorrectness statistic, *C' *= ∑_*x*,*y*_(|*g*_*x *_- *p*_*xy*_|)*d*_*xy *_which uses eqn. **2 **instead of eqn. **3**, *C' *is also equal to 0.

The inherent problem with using eqn. **2 **is more apparent if we consider *p*_*y *_= {0, 1}. This is the policy where the focal can make the greatest potential mistakes if it is unable to assess its co-player's state, where it performs the principal behaviour 50% of the time, and consequently performs the behaviour that is appropriate to its co-player's state 50% of the time. For this policy, we find that *C *= 1 and *C' *= 0.5. Now compare this to a policy *p*_*y *_= {0, 0.5}. If the focal player is again unable to assess its co-player's state, it should perform the principal behaviour 25% of the time, and will therefore be performing the behaviour that is appropriate to its co-player's state 66.7% of the time. The statistics for this policy are *C *= 0.667, and *C' *= 0.75. We see here that *C' *is therefore not a useful statistic, as it increases despite an decrease in the inaccuracy of the choice made. On the other hand, *C *accurately reports the decrease in incorrect choice-making.

### Statistic 2: S, based upon the information provided about a player's likely behaviour by knowledge of the co-player's state

The previous case defined a statistic that quantifies how dependent the actions of an individual are upon the state of its co-player by calculating the degree to which its ability to correctly respond to its co-player is reduced when it lack information about the co-player's state. Instead of using *C *(which is arbitrarily based upon the size of the maximum mistake possible), we can instead compare the uncertainty of an observer about an individual's likely behaviour, when the observer has and when it does not have knowledge of the co-player's state (we assume that in both cases the observer knows the state of the focal player). The difference between these two values, *i.e*. the reduction in uncertainty due to knowledge of the co-player's state, provides another measure of the extent to which the focal player's behaviour is influenced by the state of its co-player. This statistic relies upon quantifying the ability of an observer to predict the behaviour of a focal individual in a given state in the cases where the observer does or does not know the exact state of the co-player.

These measures are relatively straightforward to quantify. We base our definitions of uncertainty on the measure of uncertainty (or entropy) defined by Shannon and Weaver [29] (see [30] for discussion of error and noise in an evolutionary context). For an individual in state *x *with a co-player in state *y*, we can calculate an observer's Shannon-Weaver uncertainty regarding the individual's choice of action as

U(pxy)={0if pxy=0,−[pxylog⁡2pxy+(1−pxy)log⁡2(1−pxy)]if 0<pxy<1,0if pxy=1.     (5)
 MathType@MTEF@5@5@+=feaafiart1ev1aaatCvAUfKttLearuWrP9MDH5MBPbIqV92AaeXatLxBI9gBaebbnrfifHhDYfgasaacH8akY=wiFfYdH8Gipec8Eeeu0xXdbba9frFj0=OqFfea0dXdd9vqai=hGuQ8kuc9pgc9s8qqaq=dirpe0xb9q8qiLsFr0=vr0=vr0dc8meaabaqaciaacaGaaeqabaqabeGadaaakeaacqWGvbqvdaqadaqaaiabdchaWnaaBaaaleaacqWG4baEcqWG5bqEaeqaaaGccaGLOaGaayzkaaGaeyypa0ZaaiqaaeaafaqaaeWacaaabaGaeGimaadabaGaeeyAaKMaeeOzayMaeeiiaaIaemiCaa3aaSbaaSqaaiabdIha4jabdMha5bqabaGccqGH9aqpcqaIWaamcqGGSaalaeaacqGHsisldaWadaqaaiabdchaWnaaBaaaleaacqWG4baEcqWG5bqEaeqaaOGagiiBaWMaei4Ba8Maei4zaC2aaSbaaSqaaiabikdaYaqabaGccqWGWbaCdaWgaaWcbaGaemiEaGNaemyEaKhabeaakiabgUcaRmaabmaabaGaeGymaeJaeyOeI0IaemiCaa3aaSbaaSqaaiabdIha4jabdMha5bqabaaakiaawIcacaGLPaaacyGGSbaBcqGGVbWBcqGGNbWzdaWgaaWcbaGaeGOmaidabeaakmaabmaabaGaeGymaeJaeyOeI0IaemiCaa3aaSbaaSqaaiabdIha4jabdMha5bqabaaakiaawIcacaGLPaaaaiaawUfacaGLDbaaaeaacqqGPbqAcqqGMbGzcqqGGaaicqqGWaamcqqG8aapcqWGWbaCdaWgaaWcbaGaemiEaGNaemyEaKhabeaakiabgYda8iabigdaXiabcYcaSaqaaiabicdaWaqaaiabbMgaPjabbAgaMjabbccaGiabdchaWnaaBaaaleaacqWG4baEcqWG5bqEaeqaaOGaeyypa0JaeGymaeJaeiOla4caaaGaay5EaaGaaCzcaiaaxMaadaqadaqaaiabiwda1aGaayjkaiaawMcaaaaa@85AD@

Note that this measure gives a minimum value of 0 when the *p*_*xy *_is 0 or 1 (and therefore the observer can predict exactly the behaviour that will be performed), and a maximum value of 1 when *p*_*xy *_is 0.5 (and the observer is most likely to incorrectly guess the behaviour the focal individual will perform). Using eqn. **5**, the expected uncertainty of the observer when it has perfect knowledge of the co-player's state can be calculated as

U¯
 MathType@MTEF@5@5@+=feaafiart1ev1aaatCvAUfKttLearuWrP9MDH5MBPbIqV92AaeXatLxBI9gBaebbnrfifHhDYfgasaacH8akY=wiFfYdH8Gipec8Eeeu0xXdbba9frFj0=OqFfea0dXdd9vqai=hGuQ8kuc9pgc9s8qqaq=dirpe0xb9q8qiLsFr0=vr0=vr0dc8meaabaqaciaacaGaaeqabaqabeGadaaakeaacuWGvbqvgaqeaaaa@2DF7@ = ∑_*xy*_*U*(*p*_*xy*_)*d*_*xy*_.     (6)

We can calculate in a similar way the observer's uncertainty about the behaviour of the focal player, when it (the observer) does not know the state of the co-player. If the focal player is in state *x*, the probability with which it performs the principal action (averaging across all possible state values of the co-player, weighted according to their probability) is *g*_*x*_, and so the uncertainty regarding its behaviour is

U′(gx)={0if gx=0,−[gxlog⁡2gx+(1−gx)log⁡2(1−gx)]if 0<gx<1,0if gx=1.     (7)
 MathType@MTEF@5@5@+=feaafiart1ev1aaatCvAUfKttLearuWrP9MDH5MBPbIqV92AaeXatLxBI9gBaebbnrfifHhDYfgasaacH8akY=wiFfYdH8Gipec8Eeeu0xXdbba9frFj0=OqFfea0dXdd9vqai=hGuQ8kuc9pgc9s8qqaq=dirpe0xb9q8qiLsFr0=vr0=vr0dc8meaabaqaciaacaGaaeqabaqabeGadaaakeaacuWGvbqvgaqbamaabmaabaGaem4zaC2aaSbaaSqaaiabdIha4bqabaaakiaawIcacaGLPaaacqGH9aqpdaGabaqaauaabaqadiaaaeaacqaIWaamaeaacqqGPbqAcqqGMbGzcqqGGaaicqWGNbWzdaWgaaWcbaGaemiEaGhabeaakiabg2da9iabicdaWiabcYcaSaqaaiabgkHiTmaadmaabaGaem4zaC2aaSbaaSqaaiabdIha4bqabaGccyGGSbaBcqGGVbWBcqGGNbWzdaWgaaWcbaGaeGOmaidabeaakiabdEgaNnaaBaaaleaacqWG4baEaeqaaOGaey4kaSYaaeWaaeaacqaIXaqmcqGHsislcqWGNbWzdaWgaaWcbaGaemiEaGhabeaaaOGaayjkaiaawMcaaiGbcYgaSjabc+gaVjabcEgaNnaaBaaaleaacqaIYaGmaeqaaOWaaeWaaeaacqaIXaqmcqGHsislcqWGNbWzdaWgaaWcbaGaemiEaGhabeaaaOGaayjkaiaawMcaaaGaay5waiaaw2faaaqaaiabbMgaPjabbAgaMjabbccaGiabbcdaWiabbYda8iabdEgaNnaaBaaaleaacqWG4baEaeqaaOGaeyipaWJaeGymaeJaeiilaWcabaGaeGimaadabaGaeeyAaKMaeeOzayMaeeiiaaIaem4zaC2aaSbaaSqaaiabdIha4bqabaGccqGH9aqpcqaIXaqmcqGGUaGlaaaacaGL7baacaWLjaGaaCzcamaabmaabaGaeG4naCdacaGLOaGaayzkaaaaaa@7955@

The expected uncertainty for an observer with no information about the co-player's state is therefore

U¯′=∑xU′(gx)(∑ydxy).     (8)
 MathType@MTEF@5@5@+=feaafiart1ev1aaatCvAUfKttLearuWrP9MDH5MBPbIqV92AaeXatLxBI9gBaebbnrfifHhDYfgasaacH8akY=wiFfYdH8Gipec8Eeeu0xXdbba9frFj0=OqFfea0dXdd9vqai=hGuQ8kuc9pgc9s8qqaq=dirpe0xb9q8qiLsFr0=vr0=vr0dc8meaabaqaciaacaGaaeqabaqabeGadaaakeaacuWGvbqvgaqegaqbaiabg2da9maaqababaGafmyvauLbauaadaqadaqaaiabdEgaNnaaBaaaleaacqWG4baEaeqaaaGccaGLOaGaayzkaaWaaeWaaeaadaaeqaqaaiabdsgaKnaaBaaaleaacqWG4baEcqWG5bqEaeqaaaqaaiabdMha5bqab0GaeyyeIuoaaOGaayjkaiaawMcaaaWcbaGaemiEaGhabeqdcqGHris5aOGaeiOla4IaaCzcaiaaxMaadaqadaqaaiabiIda4aGaayjkaiaawMcaaaaa@4642@

Having calculated the mean uncertainties when the observer does and doesn't know the co-player's state, we can use the absolute difference between these values to determine how uncertainty changes with knowledge of the co-player's state, giving the statistic

S=U¯′−U¯.     (9)
 MathType@MTEF@5@5@+=feaafiart1ev1aaatCvAUfKttLearuWrP9MDH5MBPbIqV92AaeXatLxBI9gBaebbnrfifHhDYfgasaacH8akY=wiFfYdH8Gipec8Eeeu0xXdbba9frFj0=OqFfea0dXdd9vqai=hGuQ8kuc9pgc9s8qqaq=dirpe0xb9q8qiLsFr0=vr0=vr0dc8meaabaqaciaacaGaaeqabaqabeGadaaakeaacqWGtbWucqGH9aqpcuWGvbqvgaqegaqbaiabgkHiTiqbdwfavzaaraGaeiOla4IaaCzcaiaaxMaadaqadaqaaiabiMda5aGaayjkaiaawMcaaaaa@3720@

If there is no change in uncertainty, *S *= 0, that implies that a player's actions are completely independent of the state of its co-player. If *S *is greater than 0, this means that knowing the co-player's state allows us an observer predict the behaviour of the focal individual with a greater degree of success, which implies that the actions of the focal player are to some extent dependent upon the state of the co-player. *S *can reach a maximum value of 1, which would imply that the focal player's behaviour is completely dependent upon the state of its co-player (such that the observer can predict what action it will take with perfect accuracy when the co-player's state is known, but can do no better than guessing at random when the co-player's state is unknown).

### Comparing the statistics

**Figure 1:**
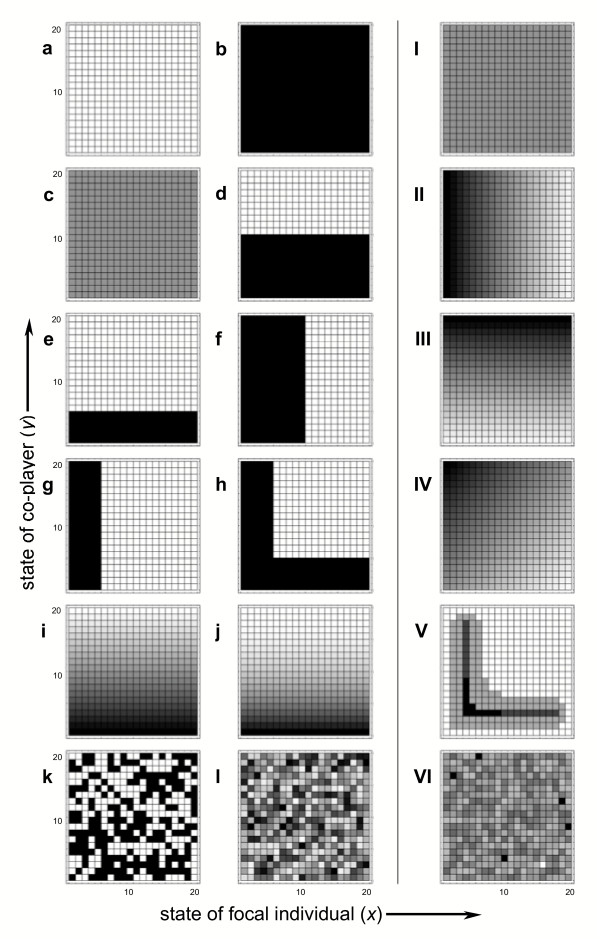
**Policy sets and distributions used as illustrations**. Policy sets used to illustrate the statistic are given in figures *a *– *l*, and distribution sets used are given in figures I – IV: see the Methods section for a full description of the policy and distribution sets. For both types of figure, the 20 × 20 squares represent the policy or distribution for focal individuals with 20 possible states (where a given individual is in state *x*) paired with co-players with 20 possible states (where a given individual is in state *y*).

Both *C *and *S *give broadly similar summaries of the degree of independence shown by individuals. Table 1 gives a number of examples based upon the policy sets and population distributions given in Figure 1 (the exact shape of each of the policies and distributions are detailed in the figure legend). Policies *a-c *are completely independent of state, and give *C *and *S *values of 0 regardless of the population distribution. Policies *d *and *e *are dependent upon the state of the co-player but not the state of the focal individual, and give differing values of *C *and *S *based upon the population distribution. Note that for a population in which pairs of individuals are distributed evenly across all state-combinations (distribution I), policy *d *gives a *C *value of 1, whilst policy *e *gives a *C *value of 0.5 – this corresponds to the fact that the best-guess of the focal individual is a 50% chance of performing the principal behaviour with policy *d*, and a 25% chance with policy *e*, which is analogous to the numerical example given above. The *S *values in *e *are greater than 0.5 however, highlighting how the two statistics can differ.

**Table 1:** *C *and *S *values for all the policies and distributions illustrated in Figure 1

	**population distribution**											
	I		II		III		IV		V		VI	
**policy**	*C*	*S*	*C*	*S*	*C*	*S*	*C*	*S*	*C*	*S*	*C*	*S*
*a*	0.000	0.000	0.000	0.000	0.000	0.000	0.000	0.000	0.000	0.000	0.000	0.000
*b*	0.000	0.000	0.000	0.000	0.000	0.000	0.000	0.000	0.000	0.000	0.000	0.000
*c*	0.000	0.000	0.000	0.000	0.000	0.000	0.000	0.000	0.000	0.000	0.000	0.000
*d*	1.000	1.000	1.000	1.000	0.524	0.830	0.750	0.950	0.812	0.966	0.931	0.994
*e*	0.500	0.811	0.500	0.811	0.143	0.371	0.312	0.620	0.699	0.922	0.493	0.800
*f*	0.000	0.000	0.000	0.000	0.000	0.000	0.000	0.000	0.000	0.000	0.000	0.000
*g*	0.000	0.000	0.000	0.000	0.000	0.000	0.000	0.000	0.000	0.000	0.000	0.000
*h*	0.375	0.608	0.286	0.464	0.108	0.278	0.187	0.385	0.476	0.613	0.368	0.591
*i*	0.526	0.318	0.526	0.318	0.319	0.237	0.423	0.294	0.459	0.296	0.494	0.310
*j*	0.411	0.369	0.411	0.369	0.203	0.333	0.307	0.368	0.464	0.348	0.401	0.357
*k*	1.000	1.000	1.000	1.000	0.870	0.981	0.932	0.994	0.900	0.989	0.890	0.987
*l*	0.462	0.231	0.469	0.233	0.441	0.236	0.455	0.236	0.447	0.224	0.437	0.233

Policies *f *and *g *are dependent upon the state of the focal player, but not the co-player, and so the focal individual's behaviour should be independent of its co-player: correspondingly, the *C *and *S *values are all 0. Policy *h *gives a typical case in which the focal player's behaviour may be dependent upon the state of both itself and its co-player, and we can see that the *C *and *S *values of this policy are intermediate between those of policies *e *and *g*. Policies *i *and *j *demonstrate that these statistics can also be used to consider policies with continuously distributed likelihoods of performing behaviours – these particular cases are dependent upon the state of the co-player but not the focal player itself, and should be compared to policies *d *and *e*. Finally, policies *k *and *l *demonstrate the sorts of *C *and *S *values expected for a randomly generated discrete and continuous policies.

**Figure 2:**
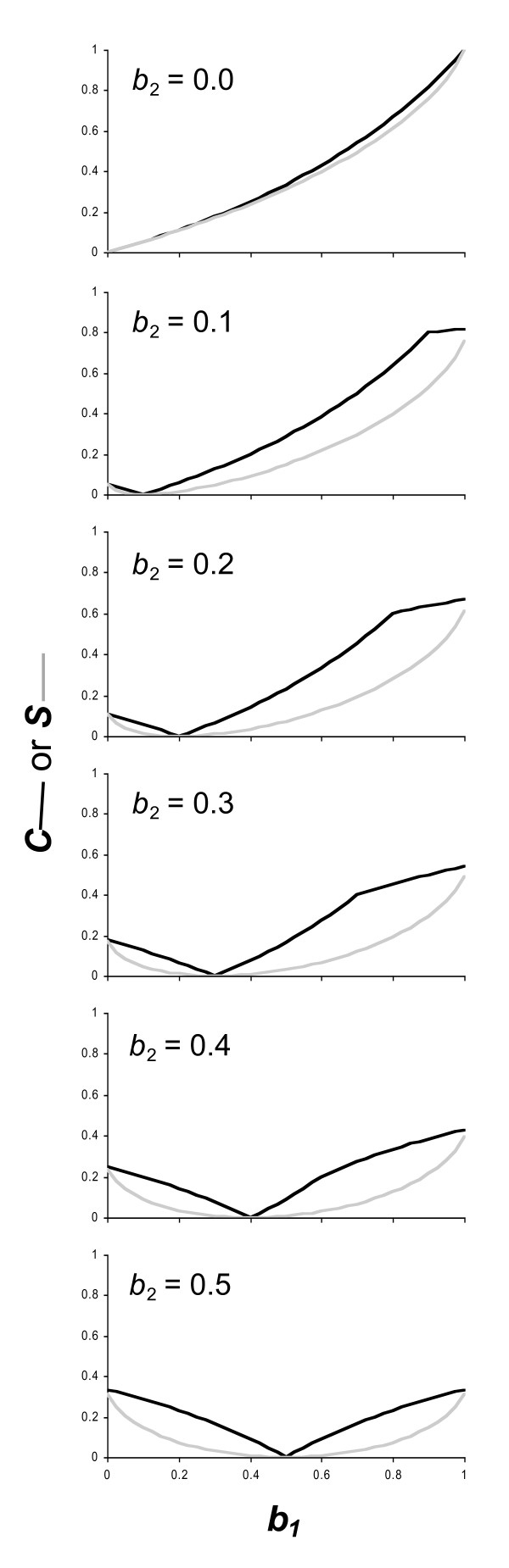
**Changes in statistics for a toy model, in responseto policy changes**. Changes in *C *(dark line) and *S *(light line) for a focal player with a single state (*x *= 1) in response to changes in the likelihood of a co-player performing the target behaviour *b*_1 _if is in the first of two possible states (*y *= 1), for the simple toy model described in the text. The graphs show changes in response to differing values of *b*_2 _(the likelihood the co-player conducts the target behaviour when it is in its second state). In all graphs *d*_11 _= 0.5 and *d*_12 _= 0.5.

In describing *C*, we used a simple example model where the focal player was always in a single state, and its co-player could be in one of two possible states. In Figure 2, we show how the values of *C *and *S *calculated for this simple example change in response to changes in the focal player's policy (represented by the changes in *b*_1 _within each panel, and *b*_2 _between each panel). This figure shows that *C *may be more sensitive than *S *to detecting small improvements in predicting how dependent an individual's actions are upon its co-player's state (shown by the more rapid increase in *C *than in *S *in the region of the graphs where *C *or *S *is close or equal to zero). This suggests that *C *may be a more useful statistic to use when it is likely that there is little dependence of players' actions on those of their co-player. *S*, on the other hand, gives a much smoother convex shape, which may be more useful at detecting small changes in dependence of action between policies where dependence is going to be high.

**Figure 3:**
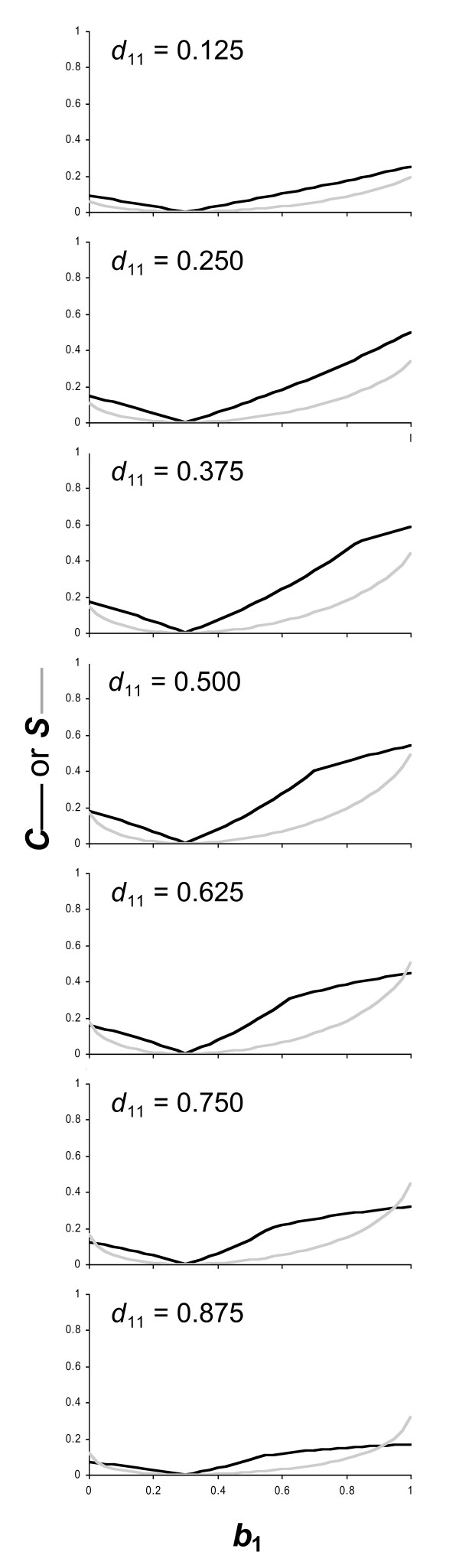
**Changes in statistics for a toy model, in responseto changes in population state distribution**. Changes in *C *(dark line) and *S *(light line) for a focal player with a single state (*x *= 1) in response to changes in the likelihood of a co-player performing the target behaviour *b*_1 _if is in the first of two possible states (*y *= 1), for the simple toy model described in the text. The graphs show changes in response to differing values of *d*_11 _(the proportion of a population of player pairs where the focal player one is in state 1 and its co-player is in state 1). In all graphs *b*_2 _= 0.3.

The panels of Figure 3 show how *C *and *S *change in response to differing probabilities of the co-player being in each of its possible states in the simple example described. This figure demonstrates that if we change the state-distribution within the population, the values of *C *and *S *will change, and the values of these statistics will be low if the state distribution is highly skewed within the population (as in the top and bottom panels of Figure 3 where *d*_11 _= 0.125 or 0.875), meaning that we should take the distribution of states into consideration if we are comparing the statistics gained for a range of different policies with separately calculated stable population distributions. Therefore, it may be sensible to combine the *S *or *C *statistic with some other measure of state distribution within the population.

In this paper, we have presented two statistical measures that both give a means of measuring how dependent the actions of a pair of players are on each other within a state-dependent dynamic game, by comparing some measurement of an individual's ability to respond appropriately to its co-player when information does or does not exist about the exact state of that co-player. In the first statistic *C*, this measure involved quantifying the change in the focal player's ability to respond correctly to its co-player, whilst the second statistic *S *quantified the change in the amount of uncertainty an observer faces in predicting the response of the focal individual to its co-player. As can be seen from these examples, *C *and *S *give broadly similar results (although they are quantitatively different), and so we leave it to the reader to decide which of these statistics they prefer to implement. We do however suggest that *S *may be more meaningful biologically:*C *is defined relative to the size of maximum mistake that an individual can make when it is in any given state, and is thus scaled to give a numerical value ranging between 0 and 1. *S *also gives a numerical value between 0 and 1, but is based upon concepts from information theory that capture how individuals use (or fail to use) the information available to them within the structure of the state-dependent policy. As described above, *C *may be more sensitive to differences in policy in situations where there is likely to be little dependence of a player's actions upon those of its co-player, whilst *S *may be more sensitive when it is very likely that there is dependence.

**Figure 4:**
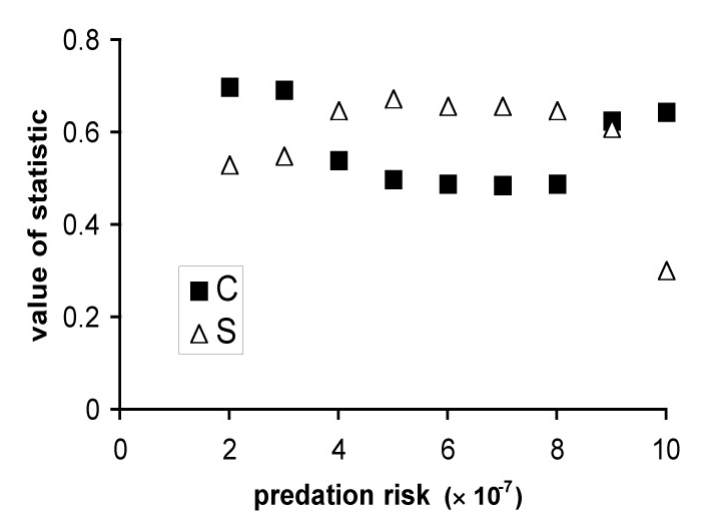
**Example of statistics being used to explore the results of a two-player dynamic game**. This example uses the forage-rest dynamic game detailed in the appendix of [17] (note that the parameter values given here purely for the purpose of illustration, and the reader is referred to this paper for an explanation of their meaning). The optimal policy and stable paired state distributions were generated for nine parameter sets, where the predation risk of foraging together *m*_*T *_(shown here as the value on the 'predation risk' axis) varies between being equal to the predation risk when resting *m*_*R *_(set here at 2 × 10^-7^, equal to the left-most value of *m*_*T*_) and being equal to the predation risk when foraging alone *m*_*A *_(set here at 10 × 10^-7^, equal to the right-most value of *m*_*T*_). This means that when *m*_*T *_= *m*_*A*_, there is no fitness advantage to an individual basing its actions upon the state of its co-player. Following the notation of [17], the other model parameters are set at *c*_*max *_= 3.0 state units, *g*_*max *_= 6.0 state units, *k *= 10^-12^, λ = 0.01, μ_*F *_= 1.5 state units, μ_*R *_= 1.0 state units, ν_1_, ν_2 _= 4.0 state units, ψ_1_, ψ_2 _= 1.0 state units, maximum state possible = 20 state units, σ_*F*_, σ_*R *_= 0.5 state units.

This is demonstrated in the output given in Figure 4, for a sample set of results from the symmetric two-player game described by Rands *et al*. [17]. In the paper, the authors note that output of the model shows the actions of the two players are relatively independent of state in cases where there is no fitness advantage to conducting an action together, but should become more dependent upon knowing the state of both players when there is some advantage to conducting a behaviour together. In Figure 4, the value of 'predation risk' given on the right-hand side of the graph corresponds to a case where there is no fitness advantage to conducting an action together; as the value of predation risk falls below this value, there is an advantage to foraging together. The values of both *C *and *S *in Figure 4 show that the quantified dependence of actions changes when the fitness advantage of conducting an action together is changed, but the statistic *S *gives a better illustration of the immediate shift from relative independence (with a value of *S *≈ 0.3 when the 'predation risk' is set at 10 × 10^-7^) to greater dependence (with a value of *S *≈ 0.6 for values of 'predation risk' lower than 10^-7^). Therefore, we suggest that *S *is a more sensitive statistic in this case, where players are likely to show a high degree of dependence of action upon the exact level each others' state.

The examples discussed above (such as the foraging game described in [17]) focus on cases where both players in the game are identical, and so the polices generated are symmetrical. However, these statistics should be especially useful in two-player games where the two contestants have different rôles (and so the strategies of the two players are potentially asymmetrical). For example, in games considering the parental care strategies of the male and female in response to each other [1,21-23], these statistics would give us a means of identifying exactly how dependent each player is on the actions of its partner. This would allow us to go beyond identifying ecological conditions where biparental care is favoured over desertion by one of the parents, and consider the degree to which partners are affected by both the actions of their partner and the ecological and life history constraints that they experience.

Similarly, in games between predators and prey (such as that developed by Alonzo [10]), we could use these statistical measures to explore the degree to which predator behaviour is determined by the state of the prey, and *vice versa*. We could, for example, use this to explore how differences in the size and energetic requirements of predators and prey affect their strategies: how do predators feeding on similarly-sized prey items differ from those that eat prey that are usually much smaller or larger than themselves? As an extreme case, we could consider the relationship between a parasite and its host as an extremely asymmetrical game (for discussion of state-dependent modelling of parasite behaviour and life history strategies, see [31,32]): parasite behaviour is likely to be extremely dependent upon host state, but would it be optimal for an infected host to respond to the state of its parasites, given that it is already infected? Using these statistical measures to quantify the degree of dependence of each player on the state of the other may yield valuable insights into the evolution of antagonistic relationships within and between species.

## Conclusion

We can see from the examples given that these techniques give useful and relatively straightforward methods of assessing the independence of an individual's action from the output generated by a state-dependent dynamic game. The statistics we describe here give us a means of quantifying the effects of changing the parameter values of dynamic game models, and consequently a means of exploring the effects of both the environment and the life history traits of the players (dependent upon the assumptions made in formulating the model). These summary statistics should be useful for assessing consensus decision making, leadership decisions, antagonistic relationships, and other situations in which there is a potential conflict of interest between individuals that base their decisions upon some aspect of the states of their group's members.

As well as giving us a means of quantifying the effects of changing parameters upon these group processes, they should allow us a means of comparing how policies and their associated stable distributions of individuals change in response to sensitivity analysis, which should contribute to our use of these models to identify behavioural rules that can then be explored both experimentally and theoretically [8,33,34].

## Methods

The statistics used in this paper are developed and described in the 'results and discussion' section above. For illustrative purposes, these statistics are used to explore the policy and distribution sets outlined in figure 1. These sets were created according to the rules detailed below.

In the policy sets, a white square represents 'perform principal behaviour with a probability of 1.0' and a black square represents 'perform principal behaviour with a probability of 0.0' (which means the alternative behaviour should be performed with a probability of 1.0). Grey squares represent the corresponding continuum between performing the principal behaviour at 0–100% of the time. Policies illustrated represent: *a*) never perform the principal behaviour; *b*) always perform the principal behaviour; *c*) perform the principal behaviour 50% of the time, regardless of state; *d*) behaviour is dependent upon the co-player's state – 'only perform the principal behaviour if *y *≤ 10'; *e*) behaviour is dependent upon the co-player's state – 'only perform the principal behaviour if *y *≤ 5'; *f*) behaviour is dependent upon personal state – 'only perform the principal behaviour if *x *≤ 10'; *g*) behaviour is dependent upon personal state – 'only perform the principal behaviour if *x *≤ 5'; *h*) behaviour is dependent upon the state of both individuals – 'only perform the principal behaviour if *x *≤ 5 or *y *≤ 5'; *i*) the probability of performing the principal behaviour is dependent upon personal state, at 1 - ((*x *- 1)/19); *j*) the probability of performing the principal behaviour is dependent upon personal state, at 1 - ((*x *- 1)/19)^2^; *k*) for any given state pair, the likelihood of performing the principal behaviour is either 0 or 1 (chosen randomly with a uniform distribution), with the additional constraint that *g*_*x *_= 0.5 for all *x*; *l*) for any given state pair, the likelihood of performing the principal behaviour is between 0 and 1 (chosen randomly with a continuous uniform distribution), with the additional constraint that *g*_*x *_= 0.5 for all *x*.

For the population distributions, shading represents the distribution of pairs within the population, with darker squares representing higher densities at a pairing. Distributions illustrated represent: I) uniform distribution of pairs across all state combinations, where *d*_*xy *_= 1/400; II) population where focal individuals are more likely to have lower state values, where *d*_*xy *_= (20 - *x*)/4200; III) population where co-players are more likely to have higher state values, where *d*_*xy *_= *y*/4200; IV) population where focal individuals are more likely to have lower state values, and co-players are more likely to have higher state values, where *d*_*xy *_= (20 - *x *+ *y*)/8000; V) state distribution similar in form to those found by Rands *et al*. [17]; VI) randomised state distribution. Note that shading is purely for illustrative purposes, and should not be compared between the distributions.

## Authors' contributions

The statistics were devised by SAR and RAJ. SAR wrote the manuscript. Both authors read and approved the final draft.
